# Clip-reinforced wrapping using the Y-shaped temporalis fascia technique for intracranial aneurysms

**DOI:** 10.3389/fsurg.2022.985240

**Published:** 2022-09-20

**Authors:** Sheng-Qi Hu, Ru-Dong Chen, Wei-Dong Xu, Jia-Sheng Yu

**Affiliations:** Department of Neurosurgery, Tongji Medical College, Tongji Hospital, Huazhong University of Science and Technology, Wuhan, China

**Keywords:** intracranial aneurysm, blood blister-like aneurysm, anterior communicating artery aneurysm, temporalis fascia, wrapping

## Abstract

**Objectives:**

This study aims to identify the effectiveness of the clip-reinforced wrapping using the Y-shaped temporalis fascia (CRYST) technique for treating intracranial aneurysms (IAs).

**Methods:**

We retrospectively reviewed five patients with ruptured IAs treated using the CRYST technique from July 2016 to May 2021. Three patients had blood blister-like aneurysms (BBAs) (one with intraoperative rupture), and two had anterior communicating artery (AcoA) aneurysms (one with intraoperative rupture). All patients had intraoperative indocyanine green angiography, and digital subtraction angiography (DSA) was reviewed 10–14 days after surgery. At 1 year postoperatively, three patients (two BBAs and one AcoA aneurysm) underwent DSA and two patients (one BBA and one AcoA aneurysm) underwent computed tomographic angiography (CTA).

**Results:**

Two aneurysms ruptured intraoperatively during the clipping, and no severe complications occurred. No patients had neurological deficits after surgery, and they had good outcomes. Four DSAs showed no aneurysms and no significant stenosis of the parent artery 10–14 days after surgery. One patient had mild stenosis of the parent artery on DSA 10 days after surgery; the stenosis improved on DSA 1 year after surgery. No other aneurysms recurred, and parent arteries were clear on CTA or DSA 1 year after surgery.

**Conclusions:**

Combining our accumulated experience in the work and literature, we described the CRYST technique to treat intractable IAs with specific morphologies and irregular wall structures in our patients. All outcomes and follow-up results were favorable.

## Introduction

Wrapping is an alternative treatment for intracranial aneurysms (IAs), which cannot be sufficiently treated using endovascular strategies or clipping. Using cotton gauze or artificial material, wrapping can induce local inflammation and generate a fibrotic scar that stabilizes the aneurysm wall (1). However, inflammatory reactions induced by wrapping materials may damage the parent vessel, leading to parent artery narrowing or occlusion (2). The perforating arteries may be damaged during the wrapping procedure, leading to brain infarction and disability. Shin et al. used the temporalis fascia and biologic glue to treat unclippable aneurysms successfully (3). Chen et al. later treated a patient with a blood blister-like aneurysm (BBA) using a Y-shaped fascia and obtained a good outcome (2). To test the appliance of this technique, we applied the clip-reinforced wrapping technique with the Y-shaped temporalis fascia (CRYST) technique in patients with BBAs and anterior communicating artery aneurysms (AcoAns) with specific configurations, particularly the intraoperatively re-hemorrhaged aneurysms. In this article, we described the procedures and technical details of CRYST techniques and showed the clinical and angiographic outcomes of the patients.

## Methods

### Patients and data

**Figure 1:**
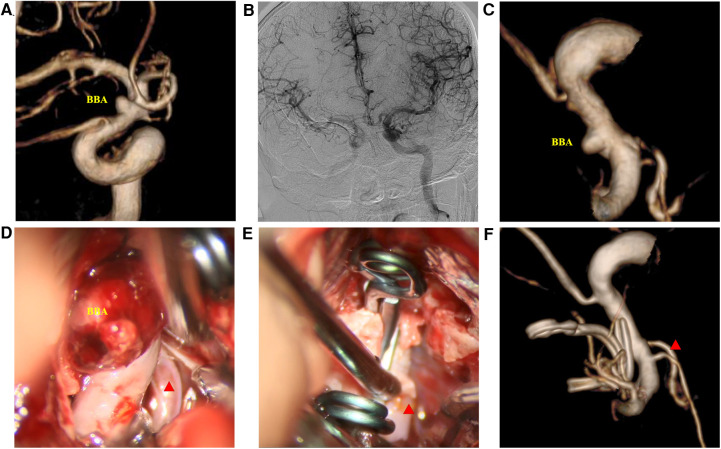
Clip-reinforced wrapping using the temporalis fascia technique for blood blister-like aneurysms. (**A**) Three-dimension digital subtraction angiography (3D-DSA) of the ICA. (**B**) Matas test revealing adequate compensation. (**C**) 3D-DSA images at the operation angle. (**D**) Microscopic view revealing the hematoma at the surface of the BBA. (**E**) Two permanent clips treating the aneurysm *via* the CRYST technique. (**F**) DSA images ten days postoperatively. Red triangle: posterior communicating artery.

Our hospital's institutional ethics committee approved this study, and we obtained the consent of the patients or their close relatives. From July 2016 to May 2021, five patients were diagnosed with ruptured IAs and underwent CRYST procedures, including three patients with BBAs and two with AcoAns. All patients underwent emergent computed tomography (CT) on admission and digital subtraction angiography (DSA) within 72 h after hemorrhage to identify morphologic features (Figures 1A,C). The surgical selection was determined by the clinical characteristics, Hunt–Hess grades, and patient willingness. Three patients with BBAs underwent the Matas test preoperatively to analyze the compensation (Figure 1B). Indocyanine green angiography was used intraoperatively in all patients. All patients underwent DSA at 10–14 days postoperatively (Figure 1F). CTA or DSA was performed 1 year after surgery. Based on the three-dimensional DSA, the following morphological parameters were measured: D1—diameter of the proximal end of the parent artery or the diameter of the anterior communicating artery (AcoA) for AcoAns; D2—diameter of the distal end of the parent artery or diameter of A2 segment for AcoAns; and PD—perforating artery diameter or the A1 segment for AcoAns.

### Surgery

**Figure 2:**
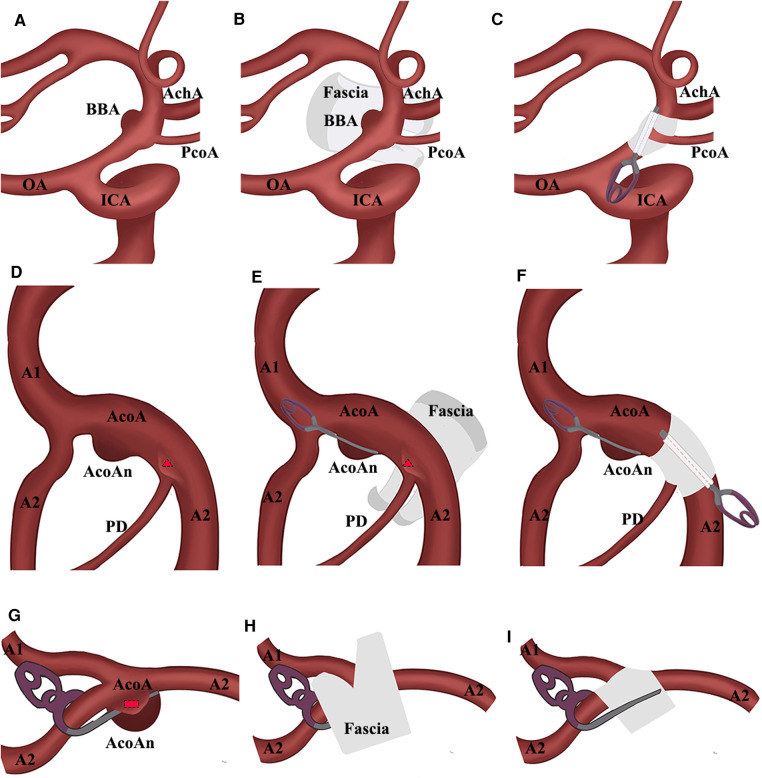
Schematic presentation. (**A**) BBA located at the supraclinoid segment of ICA; (**B**) Y-shaped temporalis fascia wrapped the BBA; (**C**) PcoA crossed through the gap of the fascia and aneurysm was disappeared; (**D**) multiple acoAns; red triangle means the microaneurysm near the ipsilateral A2; (**E**) AcoAn was simply clipped and microaneurysm was wrapped by the Y-shaped temporalis fascia; (**F**) AcoAn was simply clipped and microaneurysm was disappeared; (**G**) remnant of large AcoAn treated by the simply clipping was presented as a red rectangle; (**H**) fascia wrapped the aneurysm and A1 crossed through the gap; (**I**) AcoAn was disappeared. AchA, anterior choroidal artery; AcoA, anterior communicating artery; AcoAn, anterior communicating artery aneurysm; BBA, blood blister-like aneurysm; CRYST, clip-reinforced wrapping using Y-shaped temporalis fascia; ICA, internal cerebral artery; OA, ophthalmic artery; PcoA, posterior communicating artery; PD, perforating artery.

The patient was laid in the supine position, and a craniotomy *via* the pterion approach was performed. The scalp, temporalis muscle, and fascia were separated into a single layer. The bone flap was freed, the Sylvian fissure was exposed, and the pterygoid crest and anterior clinoid process were abraded. The cerebrospinal fluid was released to lower the intracranial pressure. Anatomical separation of the surrounding adhesive structures fully exposed the field of view and facilitated exposure of the proximal end of the parent artery for temporary clipping. Neighboring or perforating vessels were exposed, including the posterior communicating artery (PcoA) and anterior choroidal artery when treating BBAs or the A1 and A2 segments when treating the AcoA aneurysm. The fascia was based on the location of the aneurysm, size, shape, and intraoperative condition. The temporalis fascia was separated and evenly tailored into a Y-shape so that the perforating vessels would pass through the gap in the fascia. We presented the process of the CRYST technique based on the different situations in Figure 2. We carefully removed the blood clot on the surface of ruptured aneurysms (Figure 1D). The Y-shape temporalis fascia was then wrapped around the aneurysm. Finally, permanent clipping was performed (Figure 1E). An intraoperative view of treating rehemorrhaged aneurysms by the CRYST technique is shown in Supplementary Video 1.

## Results

### Patient conditions and outcomes

We included five patients; three had BBAs, and two had AcoAns; three (60.0%) were females. Patients’ age ranged from 43 to 57 years, with a mean age of 47.8 years. Three patients (60.0%) had hypertension, and two (40.0%) had hyperlipidemia. Two patients (40.0%) had a smoking history, and two (40.0%) had a drinking history. None had a history of heart disease. None had a family history of subarachnoid hemorrhage (SAH) or inherited diseases such as Marfan's syndrome. On admission, Hunt and Hess's grades were I in one patient (20.0%), II in two patients (40.0%), and III in two patients (40.0%).

Morphological parameters of the parent artery and the perforating artery are presented in Table 1. Compared with the preoperative diameters, the parent artery of BBA with intraoperative rehemorrhage had slight stenosis at 10 days and 1 year, respectively [D1, 4.1 mm vs. 3.3 mm, 3.6 mm; D2, 3.8 mm vs. 3 mm, 3.2 mm; perforating artery diameter (PD) and 2 mm vs. 1.8 mm, 1.8 mm]. However, good reconstruction could be seen after 1 year. None had complications or neurological deficits.

**Table 1:** Outcome of the IA treated using the CRYST technique.

Patient	Type	During operation	DSA			DSA			Review 1 year	Review after 1 year		
			Preoperation			10–14 days						
			D1	D2	PD	D1	D2	PD		D1	D2	PD
1	BBA	Rehemorrhage	4.1	3.8	2	3.3	3.0	1.8	DSA	3.6	3.2	1.8
2	BBA	—	3.6	3.6	1.2	3.3	3.3	1.0	DSA	3.4	3.3	1.1
3	BBA	—	3.7	3.5	1.4	3.3	3.3	1.1	CTA	3.5	3.3	1.3
4	AcoAn	Rehemorrhage	2.6	2.4	2.8	2.5	2.2	2.8	CTA	2.6	2.3	2.8
5	AcoAn	—	2.4	2.3	1.2	2.3	2.2	1.0	DSA	2.4	2.2	1.1

CRYST, clip-reinforced wrapping using Y-shaped temporalis fascia; IA, intracranial aneurysm; BBA, blood blister-like aneurysm; AcoA, anterior communicating artery; AcoAn, anterior communicating artery aneurysm; D1, proximal diameter of the parent artery or AcoA diameter for AcoAns; D2, distal diameter of the parent artery or A2 segment for AcoAns; PD, perforating artery diameter.

### Intra- and postoperative findings

**Figure 3:**
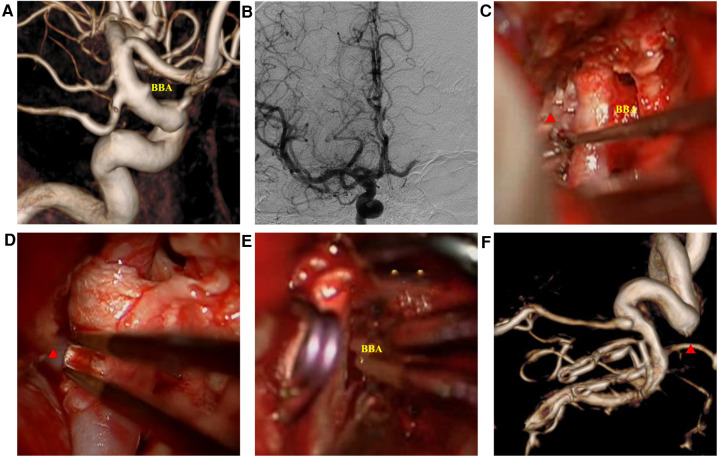
Intraoperatively ruptured blood blister-like aneurysm (BBA) images. (**A**) three-dimension digital subtraction angiography showed the BBA; (**B**) Matas test showing adequate compensation; (**C**) microscopic view showing the BBA and surrounding structures; (**D**) Y-shaped fascia preposition, posterior communicating artery (red triangle) crossing the fascia valves; (**E**) BBA neck avulsion; (**F**) DSA 14 days after the surgery revealing slight stenosis of the internal carotid artery.

**Figure 4:**
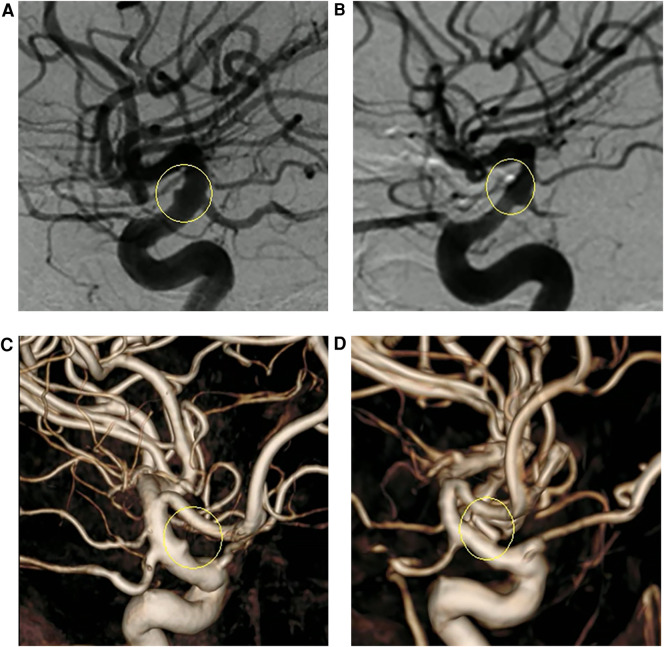
(**A,C**) Presurgical two-dimensional and three-dimensional digital subtraction angiography (DSA) images, respectively. (**B,D**) DSA images 1 year after surgery revealing that the aneurysm had not recurred, and the parent artery was well reconstructed.

Intraoperative rupture hemorrhage occurred in two patients (one BBA and one AcoAn) (Table 1). The intraoperatively ruptured aneurysms were then treated with the CRYST technique without complications. All patients underwent indocyanine green angiography (ICGA), showing that the parent artery was clear and the proximal end was visualized. Follow-up DSAs were performed 10–14 days after surgery (Figure 1F), revealing no aneurysm recurrences. One patient had mild stenosis of the parent artery (Figure 3F); however, a DSA review 1 year after surgery revealed no aneurysm, and the stenosis got improved (Figures 4B,D). Two patients (one BBA and one AcoAn) underwent CTA after 1 year, and no evident signs of recurrence were seen.

### Typical patients

Case 1: A 52-year-old woman was hospitalized with a sudden headache and vomiting for 8 h. According to the Hunt–Hess scale, the patient's condition was classified as grade II. There were signs of meningeal irritation. A CT scan revealed diffuse SAH. DSA revealed an aneurysm at the dorsal wall of the supraclinoid segment of the left internal carotid artery (ICA) (Figure 3A), suggesting a BBA. The preoperative Matas test showed that the blood flow of the right ICA had adequate compensation (Figure 3B). The BBA and its contralateral PcoA were carefully separated from the surrounding structures during the operation. As the video showed, the Y-shaped fascia was prepositioned in advance, and PcoA passed through the Y-shaped fascia gap (Figure 3D). After three temporary clips were placed to close the PcoA and the distal and proximal ICA, we tried to clamp the BBA simply. Unfortunately, the walls of the aneurysms avulsed (Figure 3E). The aneurysm was then wrapped with fascia and treated with two permanent clips. After releasing the temporary clips, the aneurysm was completely clipped, and no bleeding was detected. DSA performed 10 days postoperatively revealed that the aneurysm had disappeared, and the PcoA was clear. However, slight stenosis of the ICA was found (Figure 3F), and the patient did not have any neurologic deficits. After 1 year, DSA revealed that the BBA had not recurred, the ICA was reconstructed, and the PcoA was clear (Figures 4B,D). There were no complications during the follow-up period.

**Figure 5:**
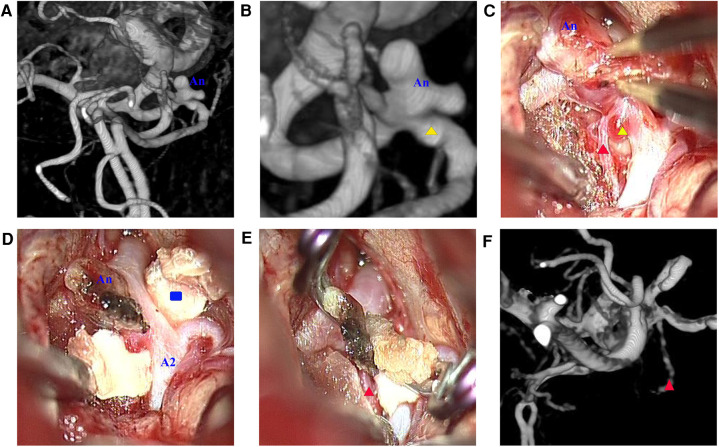
(**A**) Lobulated aneurysm (An); (**B**) magnified view revealing a microaneurysm (yellow triangle) located near the ipsilateral A2 segment of the anterior cerebral artery; (**C**) microscopic view revealing a perforating vessel (red triangle) between the lobulated aneurysm and the microaneurysm; (**D**) Y-shaped fascia (blue rectangle) was positioned; (**E**) two clips were applied to reinforce the wrapping; (**F**) digital subtraction angiography 1 year after surgery revealing that the aneurysm vanished and the perforating vessel was unobstructed.

Case 2: A 57-year-old woman was admitted to the hospital with a chief complaint of sudden headache for 6 h. An emergent CT scan revealed SAH, and DSA revealed a lobulated AcoAn with two blebs at the top (Figure 5A). A mild projection was found near the AcoAn (Figure 5B). Intraoperative images confirmed that this mild projection was a microaneurysm located near the ipsilateral A2 segment of the anterior cerebral artery (ACA) (Figure 5C). There was a perforating vessel between the microaneurysm and the lobulated aneurysm (Figure 5C). Initially, the lobulated aneurysm was treated with a miniclip. The lobulated aneurysm was obliterated. The AcoA and surrounding perforating vessels were preserved. However, this microaneurysm could not be treated. Because the thin wall presented a high risk of hemorrhage, we performed the CRYST technique. The previously placed clip was removed, and the Y-shaped fascia was prepositioned. Then, the lobulated aneurysm was clipped from the left side, and the microaneurysm was wrapped and clipped from the right side (Figure 5E). Finally, the lobulated aneurysm was completely clipped, the microaneurysm was wrapped tightly, and AcoA and perforating vessels were all preserved. A follow-up DSA at 1 year revealed that the lobulated aneurysm and microaneurysm had not recurred, and the perforating artery was clear (Figure 5F). There were no complications during the follow-up period.

## Discussion

IAs often present a challenge for surgical management and are associated with various morbidities, especially for aneurysms with specific features that were intractable to clip (4). In the acute phase of hemorrhage, endovascular therapy also carries a high risk of thrombosis (5). Wrapping is a treatment for IAs that cannot be sufficiently treated by endovascular strategies or clipping. Although Dolt's wrapping technique inspired some early studies in 1931, the muscle paste and cotton gauze have mired the technique into questions regarding the uncertainty of prevention of rebleeding and inflammation reactions (6, 7). In addition, artificial materials lacked biological activity resulting in a foreign-body granuloma and damage to perforating arteries leading to neurological deficits (2). Although Coe et al. early caused thrombosis following the use of the pieces of the temporal fascia and cyanoacrylate for wrapping the aneurysm in a patient (8), Shin et al. treated 14 cases of unclippable aneurysms by wrapping using fascia and biologic glue successfully (3). Chen et al. subsequently used Y-shaped fascia to treat a ruptured aneurysm and get a good prognosis (2). We applied the CRYST technique in some cases and got favorable results. The CRYST technique could provide sufficient uniform strength and prevent damage to the perforating arteries.

### Choice of the treatment

BBAs were considered pseudoaneurysms because they lacked an internal elastic lamina and media instead of having adventitial and fibrous tissue (9). The patient with an AcoAn also had a thin and fragile configuration. It often rebleeds intra- and postoperatively. Clinical series employed various surgical and endovascular techniques, reflecting the complexity of these aneurysms, and there is a lack of convincing evidence of the superiority of any method (10).

ICA sacrifice might not be viable in those rare patients with ICA BBAs that present with SAH (11). Furthermore, ICA sacrifice is needed for sufficient well-developed collaterals. An extracranial-to-intracranial (EI) bypass can assure the safety of ICA sacrifice. Nevertheless, bypassing the deep areas often requires an uninterrupted duration of blocking and a high level of surgical skill. Perioperative stroke rates of up to 14% highlight the intrinsically high-risk nature of bypass, with overall complication rates in complex IAs approaching 50% (12). Endovascular treatment of BBA has made substantial progress (13); nevertheless, IAs that are small or adopt strange forms present challenges. Due to dual antiplatelet therapy, there is an increased risk of subsequent surgical procedures such as external ventricular drainage or decompressive craniectomy (14).

Wrapping with routine materials (e.g., cotton, gauze, cellulose fabric, polytetrafluoroethylene, or Gore-Tex) has been cited in several studies (15–18). Wrapping with Gore-Tex is debated due to the associated recurrence rate (15). These materials are inelastic and possess even widths that cannot be fitted optimally with uneven arteries due to atherosclerosis (2). Furthermore, lack of biological activity may result in a foreign-body granuloma narrowing the parent artery, leading to neurologic deficits (18). Cotton augmentation is not reliable for treating aneurysms prone to rebleeding (17, 19). Direct suturing was criticized for wall tension, stenosis of vascular lumens, and prolonged blockage times (20, 21).

The Sundt clip graft could avoid the suture and be initially designed to repair injuries to vessel walls during the surgery (22); the technique was used to treat BBA, repair acute vessels, and decompress the trigeminal nerve (23, 24). It delivers mild mechanical stress to the surrounding nerve and could clip the dissecting aneurysms. However, the Sundt clip could lead to stenosis of the parent artery due to its unsuitable size and could injure the surrounding perforators.

ICA ligation has not been the standard option and often requires EI bypass. Braided stents, Willis-covered stents, and flow diverters (FDs) needing dual antiplatelet therapy could put patients at risk of ischemia and even hemorrhagic infarction (25, 26). FDs were associated with lower retreatment rates but higher occlusion rates (27). FDs were also prone to cause perforator occlusion and rebleeding of ruptured aneurysms, and there have even been reports of aneurysm rupture and bleeding after placing FD in the supraclinoid process segments (28).

### CRYST technique

The CRYST technique is a modification of wrapping with autologous tissue. It could protect the PcoA and AcoA. Furthermore, comparatively common technical requirements allow most institutes to perform the technique. Based on our experience, the CRYST technique has many details that must be addressed. Preoperative DSA is required to assess compensation using the Matas test. Adequate compensatory blood flow allows the time of temporary occlusion. A high-flow bypass should be prepared, although it may not be used; therefore, it is appropriate to assess the radial artery and great saphenous vein for varicose and surgical history.

The temporalis fascia should be uniformly thick during the procedure without residual muscles. It is advisable to fully wrap the base of the aneurysm with more than 2–3 mm. Exceeding the length of the blades of the clip interferes with the wrapping and exploration field after the clipping. The anterior clinoid process must be removed *via* the subdural approach in BBA patients. The distal ring should be incised, and the optic canal should be opened for proximal control before the removal of the hematoma. Most importantly, the necessity of gentle suction must be emphasized. Excessive remnant hematoma complicates clarification of the range of the aneurysm base; this phenomenon may account for inaccurate wrapping. Overly thick hematoma may cross through the IA necks and protrude retrogradely into the IA, blocking the flow and ischemia complications. Again, we emphasize the significance of prepositioning the Y-shaped temporalis fascia. The preset fascia can help to clearly identify the vessels, facilitate hematoma removal, intraoperatively adjust the clips, and prevent emergent situations like an intraoperative rupture. An electrophysiological test and ICGA were required. Although ICGA cannot visualize the parent artery near the aneurysm, it can be determined using proximal and distal imaging. The CRYST technique can only be used for small aneurysms because it requires full exposure to the surgical field, and its large size makes it difficult to separate the IA and poor wrapping effect; nevertheless, the size does not matter significantly. Endovascular therapy can be used to treat these larger aneurysms. In a sense, the CRYST technique can be complementary to interventional therapy for aneurysms with abnormal structures.

### Limitations

This study had some limitations. First, the limited sample size might reduce the robustness of our results. Nevertheless, the CRYST technique was effective for specific aneurysms, even intraoperatively and in the context of rehemorrhage. Second, two patients (one AcoA and one BBA) underwent CTA after 1 year. Tiny aneurysms cannot be clearly identified in the CTA. During the follow-up, patients did not have any discomfort and were unwilling to undergo DSA at the hospital. Further studies should consider more reliable methods. Third, a more prolonged follow-up period might improve the evidence's reliability. Finally, the locations of IAs are not generalizable; Therefore, more studies with sufficient sample sizes will be conducted in the future.

## Conclusions

Combining our accumulated experience in the work and literature, we used the CRYST technique effectively to treat intractable IAs with specific morphologies and irregular wall structures. All outcomes and follow-up results were favorable.

## Data Availability

The raw data supporting the conclusions of this article will be made available by the authors, without undue reservation.
